# The potential bactericidal effect of oral probiotics on extrinsic black stain-associated bacteria in children: An *in-vitro* and *in-vivo* study

**DOI:** 10.1016/j.jds.2025.04.032

**Published:** 2025-05-08

**Authors:** Nour Wahba, Dina Sharaf, Mohamed Wagdy, Ahmed El-Motayam, Basma Nagi

**Affiliations:** a Department of Pediatric Dentistry and Dental Public Health, Faculty of Dentistry, Ain Shams University, Cairo, Egypt; b Department of Pediatric Dentistry and Dental Public Health, Faculty of Dentistry, Alexandria University, Alexandria, Egypt; c Department of Periodontology and Oral Medicine, Faculty of Dentistry, Ain Shams University, Cairo, Egypt; d Department of Pediatric Dentistry and Dental Public Health, Faculty of Dentistry, Cairo University, Cairo, Egypt

**Keywords:** Probiotics, Black stains, Extrinsic stain, Chromogenic bacteria, Children

## Abstract

**Background/purpose:**

The extrinsic black stain (EBS) is a resistant esthetic problem in children. The aim of this study was to investigate the bactericidal effect of three probiotics strains on two types of EBS-associated bacteria *in-vitro* and *in-vivo* in children.

**Materials and methods:**

In the *in-vitro* study, two bacterial strains (*Aggregatibacter actinomycetemcomitans* and *Actinomyces naeslundii*) were revived and incubated under anaerobic or microaerophilic conditions. Cultures were grown in Brain Heart Infusion broth, adjusted to a 0.5 McFarland standard, and diluted to 1.0 × 10⁶ CFU/mL for minimum inhibitory concentration (MIC) testing. Three Probiotic strains (*Streptococcus Salivarius* BLIS M18, *Lactobacillus reuteri* LR08, *Lactobacillus paracasei* Lpc- 37) were similarly cultured and processed, and the MIC was determined using a 96-well microtiter plate, with bacterial growth assessed visually and spectrophotometrically. In the *in-vivo* study, unstimulated saliva samples were collected from patients with EBS before and after 14 days of probiotic administration. Microbial DNA was extracted and quantified using spectrophotometry.

**Results:**

In the *in-vitro* study, bacterial growth declined progressively with increasing probiotic concentrations, confirming a dose-dependent inhibitory effect. Statistical analysis showed significant reductions for both species (P < 0.001) compared to controls, with *A. actinomycetemcomitans* exhibiting a stronger response. In the in-vivo study, *A.actinomycetemcomitans* was fully eradicated, while *A. naeslundii* showed only partial suppression.

**Conclusion:**

Probiotics significantly inhibited the growth of EBS-associated bacteria in both *in-vitro* and *in-vivo* settings, resulting in the elimination of *A. actinomycetemcomitans* and a marked reduction in *A. naeslundii* count.

## Introduction

The extrinsic black tooth stain (EBS) is a distinct form of discoloration, characterized by a black line, or an incomplete coalescence of dark spots usually localized on the cervical third of teeth following the contour of the gingiva.^1^ EBS has long been recognized as a prevalent clinical and aesthetic concern,with prevalence ranging from 3.1 to 18.5 %.^2^ While more common in children, it is also seen in adults.^3^

The microbiological origins of EBS remain controversial, though multiple species have been implicated, including *Actinomyces, Prevotella nigrescens*, *Corynebacteriaceae* and the periodontal pathogen *Aggregatibacter actinomycetemcomitans (A. actinomycetemcomitans).*^4^ Notably, *Actinomyces naeslundii* (*A. naeslundii*) are frequently associated with EBS formation, likely due to their role in generating iron sulfide pigments. These insoluble compounds arise from reactions between bacterial hydrogen sulfide and iron in saliva and gingival crevicular fluid.^5^ Interestingly*, A. actinomycetemcomitans,* a well-established periodontal pathogen,^6^ not only appears at high levels in EBS,^7^^,^^8^ but was even shown to induce EBS in an ex-vivo model using sound primary teeth.^9^

Although EBS correlates with reduced caries incidence in children, suggesting possible protective effects,^3^ their cosmetic impact remains concerning. These visible stains can damage a child's self-esteem, often creating lasting psychosocial effects for both the child and their family.^4^ To date, EBS is only treated by their regular removal using professional scaling and polishing, since they aren't removed by oral hygiene practices.^2^ Unfortunately they have a high tendency of recurrence, even with routine toothbrushing.^10^

Since no evidence-based method currently exists to prevent EBS recurrence, researchers have started exploring, whether specific probiotics, particularly *Streptococcus salivarius* M18 and *Lactobacillus reuteri* might help modulate the oral microbiota and potentially prevent both EBS recurrence.^5^^,^^11^, ^12^, ^13^, ^14^ To date, only one *in-vitro* study has tested probiotics against *A. actinomycetemcomitans* and *A. naeslundii*, reporting bactericidal activity.^15^ However, no trials have assessed these effects in children's saliva.

Therefore, this study aimed to investigate the bactericidal effect of three strains of probiotics on two EBS-associated bacteria *in-vitro* and *in-vivo* in children.

## Materials and methods

### *In-vitro* study

#### Bacteria and bacterial culture

Bacterial strains of *A. actinomycetemcomitans* (NCTC 9710) and *A.naeslundii* (CDC X-600) were retrieved from frozen stocks obtained from the American Type Culture Collection (ATCC, Manassas, VA, USA).Each strain was streaked onto Brain Heart Infusion (BHI) agar with 5 % sheep blood and incubated anaerobically or micro-aerophilically at 37 °C for 48 h to revive viable colonies.^16^ A single colony from each strain was inoculated into BHI broth and incubated at 37 °C for 18–24 h under similar conditions. The bacterial cultures were adjusted to a 0.5 McFarland standard (1.5 × 10^8^ CFU/mL) using a spectrophotometer at 600 nm and diluted in fresh BHI broth to a final concentration of 1.0 × 10^6^ CFU/mL for the Minimum Inhibitory Concentration (MIC) assay.^17^

Three probiotic strains *(Streptococcus Salivarius BLIS M18, Lactobacillus reuteri LR08, Lactobacillus paracasei Lpc-37* (Super Teeth, Boise, ID, USA), were cultured in broth at 37 °C for 24 h under anaerobic conditions. The bacterial density was adjusted to match a McFarland standard (1.0 × 10^9^ CFU/mL), and the suspension was subsequently centrifuged at 10,000×*g* for 10 min to pellet the bacterial cells. The pellet was then re-suspended in sterile phosphate buffered saline, and serial dilutions were prepared in sterile BHI broth to achieve a range of probiotic concentrations, namely 0.01, 0.1, 1.0, 10, and 100 μg/mL.

#### Antimicrobial activity of the selected probiotics strains against oral bacteria

The MIC was determined using a 96-well microtiter plate following the standard Clinical and Laboratory Standards Institute (CLSI) guidelines.^18^ Each of those well plates were filled with 100 μL of BHI broth. The 100 μL of the highest probiotic concentration (100 μg/mL) was added to the first well, and a 10-fold serial dilution was performed across the plate.

After incubation, bacterial growth was assessed by visual inspection and spectrophotometric measurement of the optical density of each tube at 600 nm using TECAN microplate ELIZA reader (TECAN, Männedorf, Switzerland.) The MIC was recorded as the lowest probiotic concentration that completely inhibits visible bacterial growth, and the percentage growth inhibition was calculated for each antibiotic concentration. MIC values were reported as the mean ± standard deviation (SD) from triplicate experiments and a dose–response curve was generated to determine the MIC using the Gompertz equation representing the lowest concentration of the probiotic inhibiting microbial growth.^16^

A standard antibiotic control (amoxicillin) was included to validate the results, in addition to a blank control well, containing broth and probiotic but devoid of bacteria to check for potential interferences.

### *In-vivo* study

#### Ethics approval and enrollment of participants

This *in-vivo* study was registered at ClinicalTrials.gov under no. NCT06834815. We included children aged between 6 and 12 years presented to the outpatient clinic of the faculty of dentistry at Ain Shams University in Egypt in November 2024, diagnosed with black stains using the modified Lobene Index^19^ including those with scores of 1–3. The exclusion criteria were patients diagnosed with other stains, those who were taking antibiotics or other medications in the previous 2 weeks. Ethical approval was obtained from the Ethical Committee of Ain Shams University (FDASU-Rec IR102420), and a written informed consent was signed by the patients’ caregivers prior to the beginning of the study.

#### Pilot study and sample size calculation

An initial pilot study was conducted on three patients to estimate the required sample size, as no prior studies on this specific probiotic intervention existed. Based on the variability in the data of pilot cases, assuming the expected population standard deviation to be 2, and employing t-distribution to estimate sample size, the study would require a sample size of 7 to estimate a mean with 95 % confidence and a precision of 2. To account for a potential dropout, the sample size increased by 20 %, resulting in a final target of 9 participants.

#### Probiotics administration and saliva collection

Participants were instructed to passively dissolve a daily tablet of probiotics for 2 weeks intra orally. One mL of saliva was passively drawn from patients in 15 mL sterile falcon tube. Patients were asked not to eat or drink (except water), brush teeth, or use mouthwash at least 1 h before sample collection. Professional prophylaxis using ultrasonic scalers and polishing was done after sample collection. Samples were placed in ice immediately after collection, transferred to the lab, and stored at −80 °C.^20^

#### DNA extraction

Microbial DNA was extracted from the collected saliva samples using a QIAamp DNA Microbiome kit (QIAGEN, Hilden, Germany). Extracted DNA was stored at −80 °C for long-term storage.^20^ The DNA's quality and quantity assessment were done using spectrophotometric method. The Nanodrop spectrophotometer (Thermo Fisher Scientific Inc., Waltham, MA, USA) was initialized with a blank using 1x TE buffer. The 260/280 ratio was recorded, with an optimal range of 1.8–2.0 indicating pure DNA. Additionally, absorbance at 230 nm was measured to assess potential contamination, with a desirable 260/230 ratio of >1.8.^21^ The 16S rRNA gene regions of both bacteria were amplified using species-specific primers in a conventional PCR setup. Extracted microbial DNA was prepared for amplification.^22^

#### Sample analysis by PCR

PCR tubes were set up with the necessary reagents, including 2.5 μL of 10x PCR buffer, 0.25 μL of 5U/mL of Taq DNA polymerase, 0.5 μL of dNTP mix (10 mM), 1.5 μL of MgCl_2_ (50 mM), 1.0 μL of forward (10 μM) and 1.0 μL reverse primers (10 μM), 2 μL of purified DNA (50 ng/mL) and complete the volume to 25 μL with nuclease-free water. Consequently, the real-time cycler initial was programmed as an activation step for 5 min at 95 °C for HotStarTaq DNA Polymerase activation. Three-step cycling: denaturation for 30sec at 95 °C, annealing for 30sec at 55 °C, extension for 30sec at 72 °C, for 35 cycles, followed by final extension at 72 °C for 5min. Finally, the PCR was performed on the Biometra T-Gradient PCR cycler (Biometra, Göttingen, Germany).

For agarose gel electrophoresis, a 0.8 % agarose gel was prepared by dissolving agarose 0.8-g (Sigma Aldrich, Saint Louis, MO, USA). After electrophoresis, the DNA was visualized under UV light by UVP Transilluminator (Analytik Jena GmbH + Co, Jena, Germany). The 1 Kb DNA Ladder RT range 250–10,000 base pairs (GeneDireX Inc., Taoyuan, Taiwan) was used as marker for DNA bands.^23^

### Statistical analysis

Statistical analysis was performed using GraphPad Prism version 9. Given the normal distribution of the data, descriptive statistics (mean and standard deviation (SD)), were used to summarize bacterial growth and gene expression levels, ensuring a robust and reliable interpretation of results. In the *in-vitro* study, one-way Analysis of Variance (ANOVA) was employed, which determined whether significant differences existed between the mean bacterial growth at different probiotic concentrations. Additionally, Dunnett's multiple comparisons test was used to compare each probiotic-treated group with the untreated control. In the *in-vivo* study, the primary outcome was evaluating the change in bacterial load, measured by the difference in expression at baseline and post-treatment, using agarose gel electrophoresis, a paired t-test was applied.

## Results

### *In-vitro* study

There was a significant reduction in bacterial growth with increasing probiotic concentrations. For *A. actinomycetemcomitans*, the untreated control group had a mean bacterial count of 101 ± 1.33, while probiotic treatments at 100 mg/mL reduced it to 15.8 ± 0.42, and amoxicillin treatment resulted in the lowest count at 11.4 ± 0.97. A similar trend was observed for *A. naeslundii*, where the control group had a bacterial count of 99.3 ± 0.70, and the 100 mg/mL probiotic dose reduced it to 35.9 ± 0.65, with amoxicillin achieving the lowest count at 12.2 ± 2.71 (Table 1). The ANOVA test indicated statistically significant differences (*P* < 0.001) between groups, confirming the inhibitory effect of probiotics.

**Table 1:** Mean values of bacterial strains after treatment with serial doses of probiotics for 48 h.

Group	*A. actinomycetemcomitans* (mean ± SD)	*A. naeslundii* (mean ± SD)	Statistics *P-value*
PC “untreated”	101 ± 1.33	99.3 ± 0.70	0.122
0.001 mg/mL	96.7 ± 0.60∗	97.5 ± 1.18∗	0.354
0.01 mg/mL	85.6 ± 1.61∗	89.4 ± 1.20∗	0.031
0.1 mg/mL	68.0 ± 0.85∗	67.1 ± 1.50∗	0.420
1.0 mg/mL	30.4 ± 0.83∗	46.1 ± 1.05∗	0.0001
10 mg/mL	21.5 ± 1.28∗	40.4 ± 0.80∗	0.0001
100 mg/mL	15.8 ± 0.42∗	35.9 ± 0.65∗	0.0001
Amoxicillin	11.4 ± 0.97∗	12.2 ± 2.71∗	0.655
Statistics (ANOVA)	*P* < 0.0001	*P* < 0.0001	<0.0001

PC: Positive control “untreated bacterial strains”, ANOVA: One-way Analysis of Variances, ∗Statistical.

Significance compared to the positive control group (*P* < 0.05), F: ANOVA test value.

Further post hoc analysis using Dunnett's test validated these findings, showing statistically significant reductions (*P* < 0.001) in bacterial growth at probiotic doses of 0.01 mg/mL and higher for *A. actinomycetemcomitans* and *A. naeslundii.* However, at the lowest dose (0.001 mg/mL), the reduction was not statistically significant for *A. naeslundii* (*P* = 0.4821), suggesting that a threshold concentration is required for the probiotic effect to be effective.

**Figure 1:**
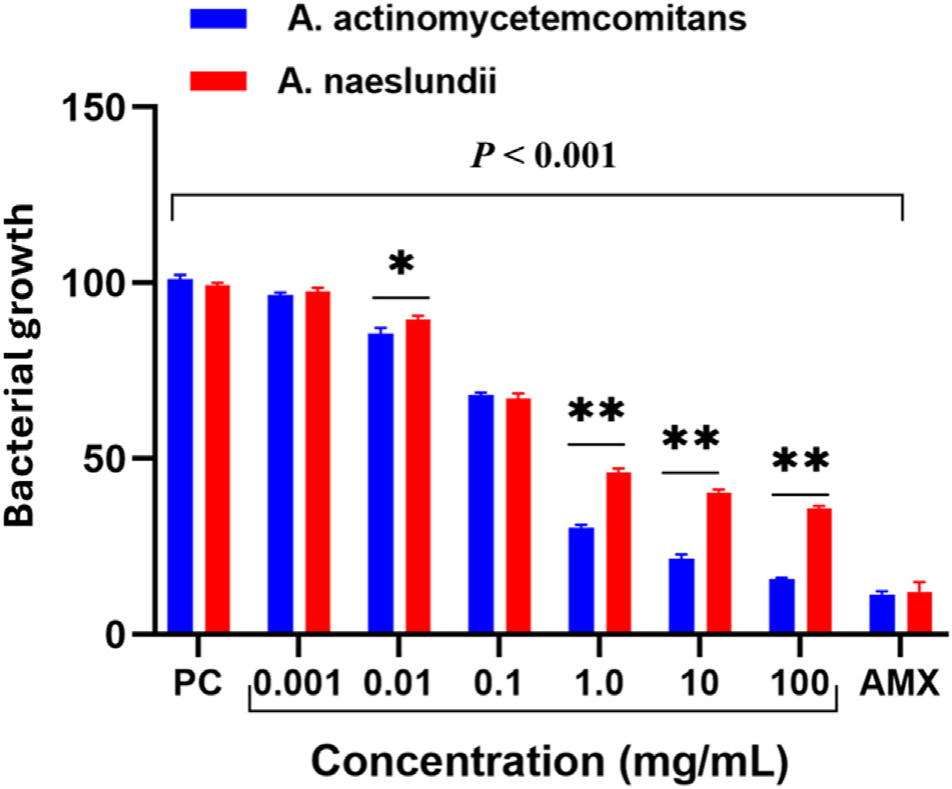
Comparison of the mean values of bacterial growth for both bacteria after treatment with serial doses of probiotics compared to untreated strains. Data are presented with mean ± SD, ∗: mild statistical significance compared to untreated strains (*P* < 0.001), AMX: Amoxicillin.

Bacterial growth declined progressively as probiotic concentration increased, confirming a dose-dependent inhibitory effect (Fig. 1). The statistical analysis indicates that for both bacterial species, reductions in growth were significant (*P* < 0.001) compared to the untreated control. However, the extent of inhibition varied between the two species, with *A. actinomycetemcomitans* showing a greater reduction in bacterial count at higher probiotic concentrations compared to *A. naeslundii*. This suggests that the former is more susceptible to probiotics, which may have implications for targeted bacterial inhibition strategies.

**Figure 2:**
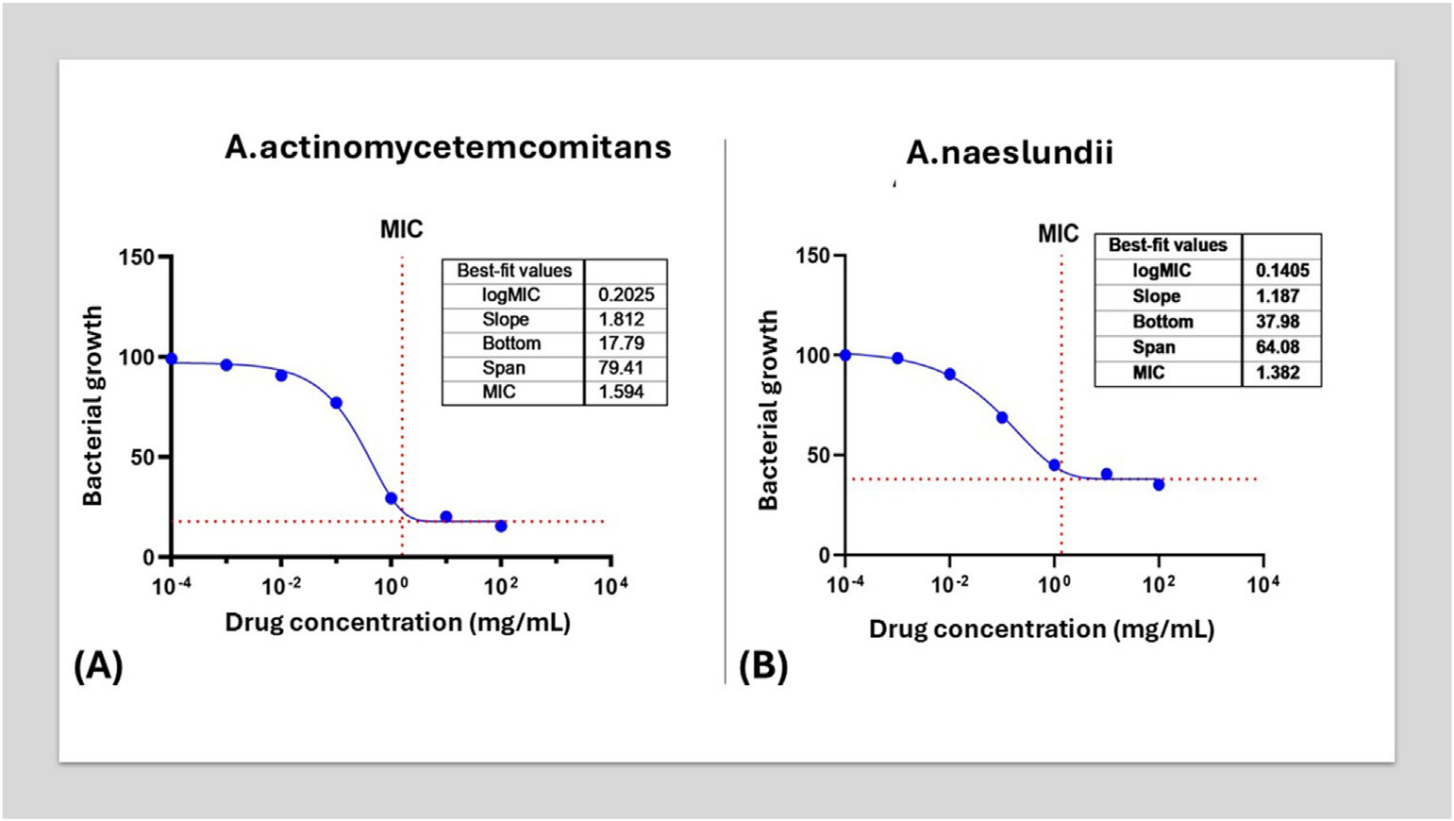
Linear regression curve illustrating the log dose of the MIC (mg/mL) of probiotics for 48 h culture of **(A)** A. actinomycetemcomitans **(B)** A. naeslundii.

The dose–response relationship of probiotics against *A.actinomycetemcomitans* and *A. naeslundii* was quantified through log-transformed MIC (mg/mL) values after 48 h of culture (Fig. 2). Linear regression analysis revealed distinct inhibition patterns: *A. actinomycetemcomitans* exhibited a best-fit MIC of 0.2025 mg/mL (logMIC = 1.812), with a steep inhibition slope (*17.79*) and maximal suppression of bacterial growth (*79.41 %*), indicating strong dose-dependent activity. *A. naeslundii* showed a lower MIC (0.1405 mg/mL; logMIC = 1.187) but a flatter slope (*37.98*) and reduced efficacy (64.08 % suppression), suggesting weaker concentration-dependent effects despite its lower threshold for inhibition.

### *In-vivo* study

**Figure 3:**
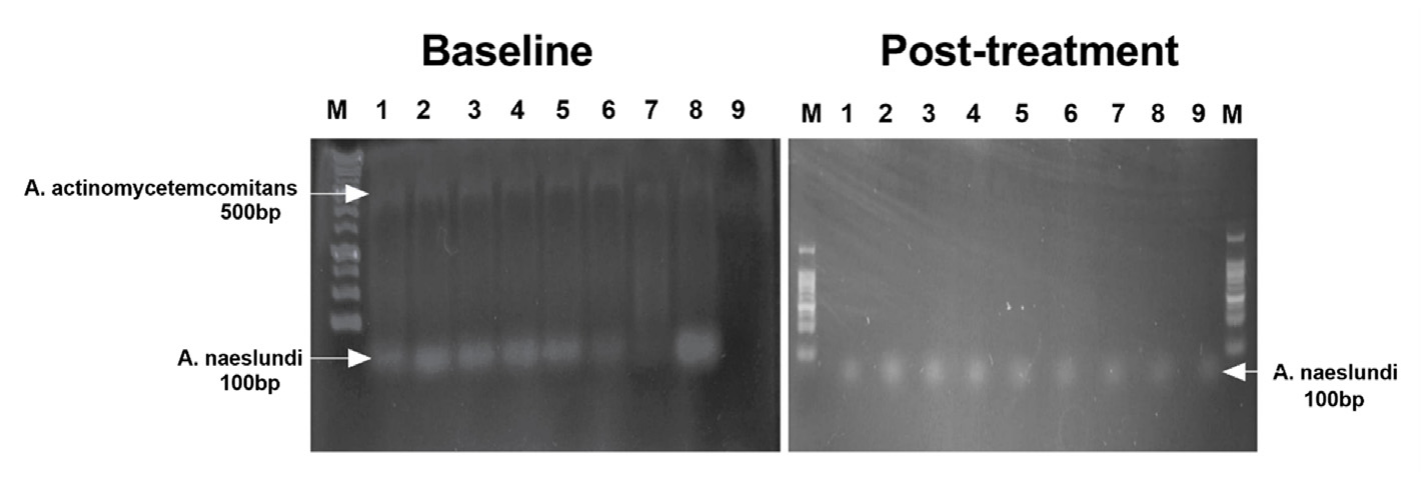
Agarose gel electrophoresis (0.8 %) of PCR-amplified 16S rRNA from microbial strains isolated from saliva samples.

Agarose gel electrophoresis (0.8 %) was used to analyze the presence of *A. actinomycetemcomitans* (500 bp) and *A. naeslundii* (100 bp) based on PCR amplification of the 16S rRNA. The observed findings are presented in Table 2 and Fig. 3 as follows.

**Table 2:** DNA band intensities for both strains at baseline and post-treatment.

Sample no	*A. actinomycetemcomitans*		*A. naeslundii*	
	Baseline	Post-treatment	Baseline	Post-treatment
1	0.55	Negative	0.85	0.52
2	0.60	Negative	0.94	0.61
3	0.54	Negative	0.84	0.53
4	0.56	Negative	0.96	0.72
5	0.64	Negative	0.84	0.51
6	0.39	Negative	0.59	0.32
7	0.22	Negative	0.12	0.11
8	0.85	Negative	0.99	0.62
9	Negative	Negative	Negative	0.27

### Baseline samples

Samples no. 1 to 8 show the presence of both bacteria indicating that they were detected in the saliva samples before probiotic treatment. Sample no. 9 was negative for both bacteria, suggesting that *A. actinomycetemcomitans and A. naeslundii* were absent or below the detection limit.

### Post-probiotic treatment

A complete eradication of *A. actinomycetemcomitans* is observed, as there is no visible 500 bp band in any lane, confirming the probiotic's strong inhibitory effect on this strain. While the intensity for *A. naeslundii* (100bp) remains at notable levels across all lanes, showing mild significant reduction. Sample no.9 showed a mild increase of *A. naeslundii* after probiotic administration.

**Figure 4:**
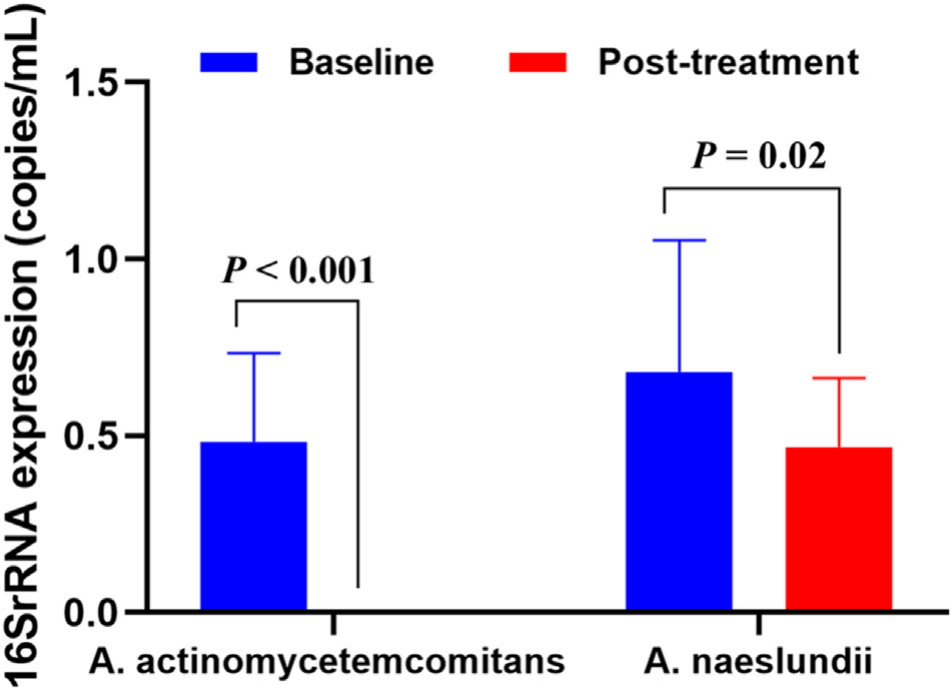
Comparison of the mean values of 16SrRNA of A. actinomycetemcomitans and the A. naeslundii at baseline and after treatment with probiotics for 2 weeks. Data are presented with mean ± SD.

A comparative analysis of the expression levels of 16S rRNA in both bacteria before and after probiotic treatment in saliva samples is illustrated in Fig. 4. A notable decrease in the expression of 16S rRNA for both bacterial species following probiotic administration, suggesting a reduction in bacterial load over time. This decrease indicates that probiotics effectively suppressed bacterial growth *in-vivo*, aligning with the *in-vitro* finding.

## Discussion

Extrinsic black stain (EBS) represents an esthetic concern in children and adults. Current management strategies rely on professional prophylaxis, which doesn't prevent its recurrence.^11^ To date, the use of probiotics for the prevention of EBS remains an underexplored strategy, prompting our interest in investigating its potential effectiveness.^12^

Probiotics, defined as live microorganisms that give health benefits when administered in adequate quantities,^24^ have gained attention in dentistry for their potential to modulate the oral microbiome and inhibit pathogenic bacteria. Clinically, they have been employed to reduce caries risk by suppressing *Streptococcus mutans*,^25^ manage periodontitis by inhibiting periodontopathogens,^26^ alleviate halitosis by targeting volatile sulfur compound-producing bacteria,^27^ and suppress oral candidiasis via competitive exclusion of *Candida albicans*.^28^

This study has evaluated the bactericidal effect of three types of probiotics against two EBS-associated bacteria, confirming their antimicrobial efficiency. Probiotics strains were selected based on their well-documented inhibitory effects against the most common EBS-associated bacteria, confirmed by previous studies.^11^^,^^12^^,^^15^^,^^29^, ^30^, ^31^

We chose to assess the effects after 2 weeks of probiotic administration because this period allows for stable colonization of probiotics strains on the tooth surfaces and competitive exclusion of EBS-associated bacteria.^32^^,^^33^ The 2 weeks duration also maintains good patients’ compliance.^34^

Our study demonstrated a significant reduction in bacterial counts of both *A. actinomycetemcomitans* and *A. naeslundi*, with the former showing greater reduction in bacterial count at higher probiotics concentration. This is in agreement with another study, which demonstrated an inhibitory effect of *Streptococcus salivarius* M18 and *Lactobacillus reuteri* strains against the same EBS-associated bacteria^15^ However, unlike their study, which tested each strain individually, our research employed a combined formulation of probiotics as provided by the manufacturer.

Our findings do not align with a recent case report by Yu et al. which noted a subtle change in *Actinomyces* level*s*.^5^ In this study, successful clinical reduction of EBS following direct topical application of a multi-strain *Lactobacillus* probiotic powder (containing *L. helveticus*, *L. pentosus*, and *L. rhamnosus*). While their study, conducted on a 41-year-old healthy male, reported a 79 % reduction in *Corynebacteriaceae*, a known EBS-associated bacterium, and notable reduction in EBS, it remains unclear, whether the observed effect was due to the probiotic strains themselves, or the mechanical abrasion caused by brushing with the probiotic powder.

Clinical evidence further supports the role of probiotics in EBS management. Bardellini et al.^11^ showed that daily Streptococcus salivarius M18 administration significantly reduced EBS recurrence in children (21.2 % vs 50 % in controls at 3 months), though the effect diminished by 6 months. Similarly, D′ Errico et al.^12^ used *Lactobacillus reuteri* strains (DSM 17938/PTA 5289) and observed marked reduction in Lobene index scores after 2 months, compared to controls. These findings support the use of oral probiotics in EBS management.

Study limitations include the small sample size and focus on only two EBS-associated bacterial species. Other studies have identified additional contributors, such *Rothia*, *Kingella*, *Neisseria*, and *Pseudopropionibacterium*, present in EBS samples of children,^31^ suggesting the need for broader microbial analyses in future work.

In conclusion, our study demonstrated that probiotics significantly inhibit the growth of EBS-associated bacteria (*A. actinomycetemcomitans* and *A. naeslundii*) in both *in-vitro* and *in-vivo* settings. We emphasize the need for further clinical trials to identify the EBS-associated microbiome across age groups, and to evaluate probiotics’ long-term efficacy in preventing recurrence, an issue with marked aesthetic and psychosocial implications.

## Declarations of competing interest

The authors have no interest relevant to this article.
